# Mannose-Modified Chitosan Poly(lactic-*co*-glycolic acid) Microspheres Act as a Mannose Receptor-Mediated Delivery System Enhancing the Immune Response

**DOI:** 10.3390/polym13132208

**Published:** 2021-07-03

**Authors:** Haibo Feng, Xiaonong Yang, Linzi Zhang, Qianqian Liu, Yangyang Feng, Daiyan Wu, Yunjie Liu, Jie Yang

**Affiliations:** 1 College of Animal Husbandry and Veterinary Medicine, Southwest Minzu University, Chengdu 610041, China; 22100128@swun.edu.cn (X.Y.); zlz754130837@163.com (L.Z.); 15892603728@163.com (Q.L.); f1733678933@126.com (Y.F.); wdx1063196822@126.com (D.W.); 2 Key Laboratory of Ministry of Education and Sichuan Province for Qinghai-Tibetan Plateau Animal Genetic Resource Reservation and Utilization, Chengdu 610041, China; 3 Department of Veterinary Medicine, Southwest University, Rongchang 402460, China; lyj_lyj1981@sina.com (Y.L.); yangyanswu@yahoo.com (J.Y.)

**Keywords:** mannose receptor, mannose-modified chitosan, PLGA nanomicrospheres, delivery system, dendritic cells

## Abstract

The mannose receptor (MAN-R)-targeted delivery system is commonly used to deliver antigens to macrophages or immature dendritic cells (DCs) to promote the efficiency of antigen presentation. To maximize the enhancement effects of chitosan (CS) and induce an efficient humoral and cellular immune response against an antigen, we encapsulated ovalbumin (OVA) in poly(lactic-co-glycolic acid) (PLGA) microspheres (MPs) and conjugated it with MAN-modified CS to obtain MAN-R-targeting nano-MPs (MAN-CS-OVA-PLGA-MPs). The physicochemical properties, drug loading rate, and immunomodulation activity of MAN-CS-OVA-PLGA-MPs were evaluated. In vitro, MAN-CS-OVA-PLGA-MPs (80 μg mL^−1^) could enhance the proliferation of DCs and increase their phagocytic efficiency. In vivo, MAN-CS-OVA-PLGA-MPs significantly increased the ratio of CD3^+^CD4^+^/CD3^+^CD8^+^ T cells, increased CD80^+^, CD86^+^, and MHC II expression in DCs, and improved OVA-specific IgG, IgG1, IgG2a, and IgG2b antibodies. Moreover, MAN-CS-OVA-PLGA-MPs promoted cytokine (IFN-γ, IL-4, and IL-6) production in mice. Taken together, our results show that MAN-CS-OVA-PLGA-MPs may act by activating the T cells to initiate an immune response by promoting the maturation of dendritic cells and improving their antigen presentation efficiency. The current study provides a basis for the use of MAN-CS-OVA-PLGA-MPs as an antigen and adjuvant delivery system targeting the MAN-R on the surface of macrophages and dendritic cells.

## 1. Introduction

Chitosan (CS), also known as deacetylated chitin, is prepared by deacetylation of chitin. CS is an alkaline polysaccharide extracted naturally, which is simple to prepare, widely available, and cheap. Pure CS is a translucent white powder that is very difficult to dissolve in water and acid and can be completely absorbed by the body [1]. It has good biocompatibility, anti-inflammatory, antibacterial, antioxidation, blood lipid and glucose reduction, immune regulation, and other biological activities [2,3,4,5]. As a natural nontoxic adjuvant, CS not only protects the activity of the antigen but also enhances the immune response of immune cells to the antigen. CS plays an effective regulatory role in the immune system of animals and humans [6]. Compared with traditional adjuvants, CS has a better vaccine protection effect. Its stable electrostatic binding ability prevents the vaccine from being enzymolyzed before reaching the target cells and maintains the integrity and stability of the original structure of the antibody, and this combination will produce an improved immune effect compared with the direct application of the vaccine [7]. Wang et al. found that the addition of CS as adjuvant to a live attenuated influenza vaccine improved its safety and its effectiveness to resist an influenza virus attack due to higher levels of secreted antibody [8,9]. Lubben et al. found that CS as an immune adjuvant played a role in facilitating a good sustained immune response and prolonged the drug effectiveness in cells [10,11].

Poly(lactic-co-glycolic acid) (PLGA) is the product of the copolymerization of lactic acid and hydroxyacetic acid. Because these two substances occur naturally and are easily metabolized by the Krebs cycle [12,13,14], PLGA is considered a biodegradable polymer and is approved by the Food and Drug Administration (FDA) and officially included in the United States Pharmacopoeia as a pharmaceutical excipient. It is characterized by high safety and has a controllable drug release rate. It is the most studied biodegradable material in biomedical engineering. The preparation and application of various PLGA drug microspheres (MPs) have been widely reported, including their application as carriers of protein and enzyme drugs [15,16].

Nanoparticles (NPs) can be prepared from natural or synthetic polymers and can be used to transport a variety of drugs. PLGA NPs very effectively encapsulate antigens and adjuvants. NPs are promising delivery systems for therapeutic agents since they can be designed to slip between intercellular spaces, enter cells, or translocate directly through biological barriers to access targeted sites. By designing modified NPs, drug release can be controlled in target organs or cells [17]. PLGA can form nanoparticles that encapsulate and continuously release a variety of antigens and drugs in biological environments [18,19]. Luo et al. prepared Chinese yam polysaccharide PLGA (CYPP) NPs in an OVA vaccine formulation and investigated their immune enhancement effect in vitro and in vivo. Their results suggested that the vaccine formulation composed of OVA encapsulated in CYPPs induced the strongest antigen-specific immune responses. The results indicated that CYPPs showed a strong immunoenhancement activity [20].

The mannose receptor (MAN-R), a subtype of the carbohydrate-recognition domain (CRD) type I transmembrane receptors with five domains, is a member of the C-type lectin superfamily [21]. MAN-Rs are highly expressed on the surface of macrophages, immature dendritic cells (DCs), and other immune cells. The MAN-R can internalize mannosylated protein molecules at high speed and deliver the ligand to the MHC-expressing cells. This process can lead to a 100-fold improvement of the efficiency of antigen presentation to T helper cells (Th cells) [22]. Therefore, a MAN-modified antigen presentation system may improve the antigen internalization efficiency of DCs and macrophages, improve the targeting effect, and reduce the side effects of vaccines [23,24]. Copland et al. investigated the effects of MAN-modified liposomes on FITC-OVA uptake, DC maturation, and T cell proliferation. The results demonstrated that when DCs were cocultured with MAN-modified liposomes containing FITC-OVA, the expression of MHC-II, CD80^+^, CD86^+^, and CD83^+^ was significantly increased [25,26]. Wi et al. synthesized MAN-labeled PLGA NPs (MAN-PLGA-NPs) encapsulating tumor-specific antigens for targeted delivery to MAN-Rs on DC surfaces. The MAN-PLGA-NPs led to highly mature and activated DCs, triggering activation of cytotoxic CD8+ T cells in tumor-bearing mice. This MAN-R-targeted delivery system promoted the delivery of tumor-specific antigens to DCs, providing a theoretical basis for the use of MAN-PLGA-NPs in cancer immunotherapy [27]. Zhu et al. synthesized MAN-modified PLGA and prepared MANNP (MNP)-loaded hepatitis B surface antigen (HBsAg) protein. MNPs displayed enhanced internalization in bone marrow-derived dendritic cells (BMDCs) and RAW 264.7 cells in vitro. MNPs can induce humoral immune and strengthened cellular immune responses in vivo. The results demonstrated that the MNPs are a promising delivery system for the hepatitis B vaccine [28].

In the present study, in order to investigate the enhancement effects of CS on the drug delivery of PLGA-MPs, we encapsulated OVA in PLGA-MPs and conjugated it with MAN-modified CS to obtain MAN-R-targeting nano-MPs (MAN-CS-OVA-PLGA-MPs). The physicochemical properties and the immunoenhancement were investigated by determining their effect on phagocytic activity of DCs in vitro and the humoral and cellular immune response in mice in vivo. Our findings provide a theoretical basis for the use of MAN-CS-OVA-PLGA-MPs as a novel adjuvant and antigen delivery system.

## 2. Materials and Methods

### 2.1. Reagents and Antibodies

Chitosan, with an MW of 600 kDa and a deacetylation degree of 85%, was procured from Sigma Chemical Co. (St Louis, MO, USA). Polyvinyl alcohol (PVA) with a purity of 98.0–98.8% was purchased from Fisher Scientific Co., Ltd. (Brussels, Belgium); DMEM (high glucose) complete medium (4.5 g/L D-glucose, sodium pyruvate, L-glutamine, phenol red, 10% FBS (0500), and 1% penicillin/streptomycin) was purchased from Shanghai Zhongqiaoxinzhou Biotechnology Co., Ltd. (Shanghai, China); The CCK-8 kit was purchased from Biyuntian Biotechnology Co., Ltd. (Shanghai, China); PLGA (MW = 10,000–20,000), pancreatin, and FITC were purchased from Shanghai Yuanye Biology Co., Ltd. (Shanghai, China); fluorescently labeled anti-mouse monoclonal antibodies (anti-CD80-PE, anti-CD40-FITC, anti-CD86-PE, anti-MHCII-PE, anti-CD4-FITC, anti-CD3-Percp, and anti-CD8-PE) were obtained from Thermo Fisher Scientific (Asheville, NC, USA).

### 2.2. Synthesis of Mannose-Modified Chitosan PLGA Microspheres

#### 2.2.1. Synthesis of Mannose-Modified Chitosan

**Figure 1:**
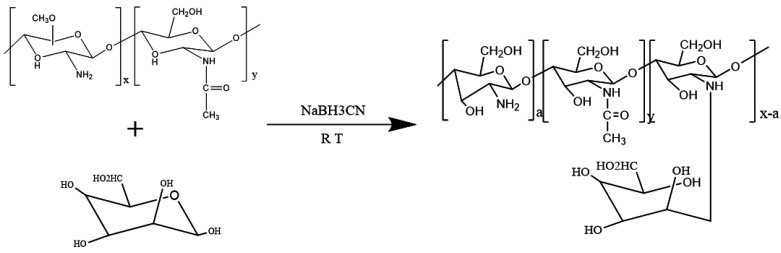
Schematic diagram of synthesizing the mannose-modified chitosan.

The MAN-modified CS was synthesized by reductive amination with minor modifications [24]. Briefly, CS was dissolved in a 1% acetic acid aqueous solution (pH = 6.5) under stirring and to produce a viscous solution. Subsequently, the MAN (mol/GlcN = 1) and sodium cyanide borohydride were added into the viscous solution. The reaction mixture was stirred at room temperature for 36 h, dialyzed against distilled water for 4–6 days, and lyophilized using a vacuum lyophilizer (Shuangjia, SJIA-18N, Ningbo, China) to produce MAN-modified CS. The synthesizing Schematic diagram of MAN-CS was illustrated in Figure 1. The chemical properties of MAN-modified CS were determined by Fourier transform infrared (FT-IR). 

#### 2.2.2. Physicochemical Property Analysis of MAN-CS

The organic functional groups of MAN-CS were determined by Fourier transform infrared (FT-IR) spectrometer (FTIR-8400S, Shimadzu Co., Kyoto, Japan), and the IR characteristic wavelengths between 4000 and 400 cm^−1^ were detected. The mannose, CS, and mannose-modified CS were dried and ground with KBr powder and pressed into pellets for FT-IR measurement.

#### 2.2.3. Synthesis of PLGA Microspheres

PLGA (50 mg) was dissolved in 2 mL of dichloromethane and placed in an ice bath. Then, 500 μL of an OVA solution (0.08 mg/μL) was slowly added dropwise into the PLGA dichloromethane solution under high-speed agitation using a homogenizer and emulsified for 60 s at 7552× *g* to form an emulsion. The emulsion was added to 20 mL of a 2% PVA solution in an ice/water bath, emulsified for 60 s at 7552× *g* forming a double emulsion, and stirred with a magnetic stirrer for 4 h to volatilize the dichloromethane. The double emulsion was centrifuged at 16,992× *g* for 10 min at a constant temperature of 4 °C and then washed three times with deionized water to produce OVA-loaded PLGA-MPs.

#### 2.2.4. Determination of Drug Loading on Microspheres

To determine the loading efficiency (LE), 10 mg PLGA-MPs was dissolved in 1 mL acetonitrile, and the solution was centrifuged at 30,208× *g* for 10 min. The supernatant was discarded, and the precipitated protein was dispersed in a PBS solution. After centrifugation, the supernatant was retained, precipitated, and dissolved in a 0.1 M NaOH solution by vortexing; then, it was combined with supernatant, and the pH was adjusted to 7 with 0.1 M HCI. The OVA content was determined by the micro-BCA method in triplicate. The LE was calculated as follows:

(1) $$\mathrm{Loading}\;\mathrm{Efficiency}\;(\%)=\frac{\left(\mathrm{Total}\;\mathrm{amount}\;\mathrm{of}\;\mathrm{OVA}—\mathrm{Free}\;\mathrm{amount}\;\mathrm{of}\;\mathrm{OVA}\right)}{\left(\mathrm{weight}\;\mathrm{of}\;\mathrm{MPs}\right)}\;\times \;100\%$$

#### 2.2.5. Orthogonal Experiment Optimization

To obtain the optimal formulation of OVA-PLGA-MPs, an L9 (3^3^) orthogonal table was used to investigate the effects of OVA dosage, PVA concentration, and PLGA dosage on PLGA-MPs. (Table 1).

#### 2.2.6. Synthesis of Mannose-Modified Chitosan PLGA Microspheres

PLGA (50 mg) was dissolved in 2 mL of dichloromethane in an ice bath. The protein solution (40 mg of OVA dissolved in 500 μL deionized water) was added dropwise to the PLGA dichloromethane solution under high-speed shearing with a homogenizer (Xinzhi, Ningbo, China). The solution was emulsified at 7552× *g* for 60 s to form an emulsion. The emulsion was transferred to 20 mL of a 2% PVA acetic acid solution in an ice/water bath, and 400 mg of MAN-modified CS (CS was added to synthesize CS-OVA-PLGA-MPs) was added and emulsified at 7552× *g* for 60 s to form a double emulsion, which was stirred using a magnetic stirrer (Shanghai Lichen, China) for 4 h until the organic solvent had evaporated. The prepared composite emulsion was centrifuged at 16,992× *g* for 10 min at 4 °C, and the sediment was washed three times with deionized water and then lyophilized to obtain MAN-CS-OVA-PLGA-MPs.

#### 2.2.7. Microsphere Surface Morphology, Particle Size, and Zeta Potential

The morphology of the MPs was observed by scanning electron microscope (Jeol, JSM-7500F, Tokyo, Japan) after spraying with gold particles. The MPs were redistributed in distilled water. The particle size and the zeta electromotive force of the MPs were measured using a laser particle sizer (Anton Paar, Litesizer 500, Vienna, Austria).

### 2.3. *In Vitro* Experiments

#### 2.3.1. Cytotoxicity of MAN-CS-OVA-PLGA-MPs on DCs

The DC single-cell solution was prepared as previously reported [29]. The DC suspension was adjusted to 1 × 10^6^ cells/mL and seeded onto a 96-well plate. Once the DCs grew into monolayers, 100 μL of different concentrations (10, 20, 40, 80, 160, or 320 μg/mL) of FITC-OVA, OVA-PLGA-MPs, CS-PLGA-MPs, or MAN-CS-PLGA-MPs at were added to the wells and cultured for a further 24 h. Next, 10 μL of Cell Counting Kit-8 (CCK-8) reagent was added to each well and the OD was measured using a microplate reader at 450 nm after 4 h.

#### 2.3.2. Phagocytosis of Different Microspheres by DC Cells

The DCs were inoculated into a 6-well cell-culture plate with a round coverslip. After 24 h of culture, FITC-OVA, CS-OVA-PLGA-MPs, or MAN-CS-OVA-PLGA-MPs were added, and after a further incubation of 12 h, the DCs on the coverslips were fixed with 4% paraformaldehyde for 20 min and permeabilized with Triton X-100 for 4 min. Next, the cells were stained with Phalloidin-iFluor 555 Reagent for 50 min and with DAPI staining solution for 5 min. Finally, the DCs were mounted with 90% glycerol and visualized using a confocal laser scanning microscope (LSM 800, Zeiss, Oberkochen Germany).

#### 2.3.3. The Effect of MAN-CS-OVA-PLGA-MPs on the Delivery of OVA

The DCs were inoculated in a 6-well cell-culture plate on a round coverslip. After 24 h of culture, FITC-OVA, CS-OVA-PLGA-MPs, or MAN-CS-OVA-PLGA-MPs were added and the cells were cultured at 37 °C for an additional 12 h. Then, the culture media were withdrawn and discarded, the DCs were washed three times with PBS, and 100 μL SDS cell lysis buffer was added into each well to lyse the cells. The fluorescence intensity (excitation wavelength 495 nm, emission wavelength 528 nm) was measured by a fluorescent enzyme labeling instrument (iMark, Bio-Rad, Philadelphia, PA, USA) to assess the cellular uptake of the different MPs.

### 2.4. *In Vivo* Experiments

#### 2.4.1. Mice Grouping and Immunization

Five-week-old Institute of Cancer Research (ICR) mice (Grade II, females, weight: 18–22 g) were provided by Sichuan Laboratory Animal Center (China). All animal procedures were performed as per internationally accepted principles, mentioned in the Guidelines for Keeping Experimental Animals issued by the government of China and approved by the Institutional Animal Care and Use Committee, Southwest University (IACUC Approval No. IACUC-20191224-18). The mice were randomly divided into 5 groups of 22 mice each. All mice were fed separately. MPs were dispersed in normal saline, and the mice were immunized with MAN-CS-OVA-PLGA-MPs (806 μg/0.2 mL), CS-OVA-PLGA-MPs (769 μg/0.2 mL), OVA-PLGA-MPs (633 μg/0.2 mL), OVA (50 μg/0.2 mL), and normal saline (0.2 mL). The mice were injected subcutaneously into the neck and tail (0.1 mL twice every week with a total dose of 0.2 mL per week). The mice were randomly selected and their blood was collected at 14, 21, 28, 35, and 42 days after the first immunization. The serum samples were separated and stored at −70 °C. The mice were sacrificed, and their spleens were collected aseptically.

#### 2.4.2. Immunophenotype of Spleen Lymphocytes in Mice

On the 28th day after the first immunization, the ICR mice were sacrificed, and the spleen was removed aseptically. A single-cell suspension was prepared according to our previous report [30]. The total number of spleen cells was about 1 × 10^6^ in a 1.5 mL EP tube, centrifuged at 265.5× *g* and 4 °C for 5 min. Next, 1 mL of 5% skimmed milk was added to block for 1 min, followed by centrifugation at 265.5× *g* at 4 °C for 5 min. The supernatant was discarded, and the cells were resuspended in 100 μL PBS, followed by the addition of 100 μL PBS containing anti-CD4-PE (1:200), anti-CD8-PE (1:200), or anti-CD3e-FITC (1:200), and the suspensions were mixed and kept, away from the light, for 30 min at 4 °C. Next, 500 μL PBS was added and mixed, followed by centrifugation at 118× *g* to remove the supernatant. Thereafter 500 μL PBS was added and mixed, and the mixture was passed through a 200-mesh nylon filter, transferred into a flow tube, and placed in an icebox. Fluorescence-activated cell sorting (FACS) (BD Biosciences, California, USA) was used to evaluate the percentages of CD3^+^CD4^+^ and CD3^+^CD8^+^ T cells present in the stained cells.

#### 2.4.3. The Maturation of DCs in Mouse Spleen

The ICR mice were immunized and were subsequently sacrificed after 24 h. Their spleens were taken out aseptically, and a single-cell suspension was prepared as previously reported [31]. Spleen cells (1 × 10^6^ in 1.5 mL) were placed in an EP tube and centrifuged at 265.5× *g* and 4 °C for 5 min. Skimmed milk (5% *m*/*v*) was added to block for 1 min, followed by centrifugation for 5 min at 265.5× *g* and 4 °C, and the supernatant was discarded. The cells were resuspended in 100 μL PBS, and then 100 μL PBS containing CD11c-FITC, CD80-PE, CD86-PE, or MHC-II-PE (all at 1:200) was added into each tube. The cells were mixed by vortexing and kept, away from light, for 30 min at 4 °C. Next, 500 μL PBS was added, mixed by vortexing, and centrifuged at 118× *g*. To the supernatant, 500 μL PBS was added; the mixture was vortexed, passed through a 200-mesh nylon filter, transferred to a flow tube, and placed on ice, and the CD11c-CD80, CD11c-CD86, and CD11c-MHC-II were determined by flow cytometry.

#### 2.4.4. Determination of OVA-Specific IgG and IgG Isotype in Serum of Mice

Indirect ELISA was used to determine the level of antibody subclass in the serum of mice 42 days following the first immunization. OVA-specific IgG titer and subclass were determined by ELISA assay as described in our previous report [32]. Briefly, 100 μL of coating solution (0.05 mol/L carbonate buffer containing 10 μg/mL OVA) was added to the wells of a 96-well enzyme plate and incubated at 4 °C for 18 h. The solution was removed, and the wells were washed 3 times for 3 min using wash buffer (0.01 mol/L PBS, 0.5% Tween-20, pH 7.2). Next, 300 μL of a 1% skim milk solution was added to each well and incubated at 37 °C for 1 h. Subsequently, the solution was removed, and the wells were washed 3 times for 3 min each time. Diluted (1:100) serum sample (100 μL/well) was added and incubated at 37 °C for 1 h. The solution was withdrawn and the wells were washed 3 times for 3 min each time. Horseradish peroxidase (HRP)-conjugated antibodies against IgG, IgG1, IgG2a, or IgG2b (1:1000 dilution) were added at 100 μL/well, and the 96-well plate was shaken to mix the solution fully, followed by incubation at room temperature for 1 h. The solution was withdrawn and the wells were washed 3 times for 3 min each time. Finally, 3,3′,5,5′-tetramethylbenzidine (TMB) substrate solution was added (100 μL/well) to develop the colorimetric reaction, followed by incubation at 37 °C in the dark for 15 min, after which 50 μL 2 M H_2_SO_4_ was added to each well to terminate the reaction. The OD450 value was determined using a microplate reader (Model 680, Bio-Rad, Philadelphia, PA, USA).

#### 2.4.5. Detection of Cytokines in Serum of Immunized Mice by ELISA

The concentration of cytokines in the serum of ICR mice was measured 42 days following the first immunization. The levels of IL-2, IL-4, IL-6, and IFN-γ in serum samples were detected by ELISA according to the instructions of the commercial kit.

### 2.5. Statistical Analysis

Test results are expressed as the mean ± standard deviation. SPSS 22 software (SPSS, Chicago, IL, USA) was used for data analysis. Duncan and LSD’s multiple range tests were used to determine the difference among groups. *p* < 0.05 was considered to be significantly different.

## 3. Results

### 3.1. Physicochemical Characteristics

#### 3.1.1. Identification of Mannose-Modified Chitosan

The MAN-modified CS was synthesized by reduction and ammoniation reaction of CS and MAN. If the synthesis of MAN-modified CS is successful, the theoretical elemental value of H and O will increase, while the elemental value of C and N will decrease [33]. The results of the elemental analysis, as illustrated in Table 2, indicate that, compared with CS, the elemental value of H and O of MAN-modified CS had increased compared with CS alone. In contrast, the elemental value of C and N had decreased. Thus, the changes in the actual values obtained by the elemental analyzer were consistent with a successful synthesis of MAN-modified CS.

#### 3.1.2. FT-IR Spectroscopy Analysis

**Figure 2:**
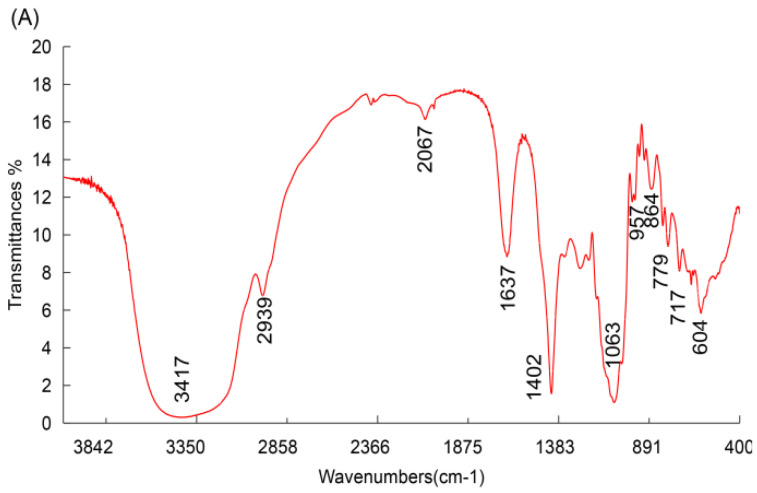

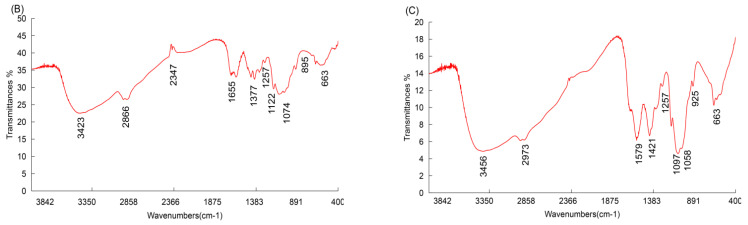
FT-IR spectra of mannose-modified CS. The IR spectrum of the sample powder was analyzed using the potassium bromide pellet method in an IR spectrophotometer. The data were determined between 4000 and 400 cm^−1^. The IR characteristics of mannose (MAN) (**A**), CS (**B**), and MAN-modified CS (**C**) are illustrated.

The FT-IR spectra of the MAN, CS, and MAN-modified CS were measured in the 400 to 4000 cm^−1^ range, and the results are shown in Figure 2A–C. The FT-IR spectrum of MAN-modified CS contains all the characteristic peaks of CS, including a broadly intense stretched peak in the 3200 to 3600 cm^−1^ region corresponding to hydroxyl and amino absorption peak stretching vibrations, and the characteristic absorption peak of methyl and methylene stretching vibrations was detected in the 2993 cm^−1^ region. An absorption peak between 1500 and 1600 cm^−1^ represented C=O asymmetric stretching vibrations. Within the 900 to 1200 cm^−1^ region, the C–O–C stretching vibration absorption represented the peaks of glucose. In addition, the absorption peaks in the 1257, 1151, 1097, 1058, 925, and 663 cm^−1^ regions represented the same characteristic stretching vibrations as seen in CS. The other absorption peaks, in the 1579 and 1421 cm^−1^ regions, represented two stretching vibrations characteristic of mannose. The results show that the polymer is composed of CS and MAN, suggesting that the synthesis of MAN-modified CS was successful [33].

### 3.2. Orthogonal Design of PLGA Microspheres

According to the orthogonal experiment design (Table 2 and Table 3), the optimal conditions for the preparation of PLGA-MPs were 40 mg PLGA, 50 mg OVA, and a concentration of PVA in the external water phase of 2%; therefore, we prepared the MPs according to this optimal formula. The particle size and drug loading capacity of the nanoparticles indicate that the MPs prepared by the method adopted in this paper display a good particle size, drug loading reproducibility, and stability.

### 3.3. Morphology of the Microspheres Obtained by SEM

**Figure 3:**
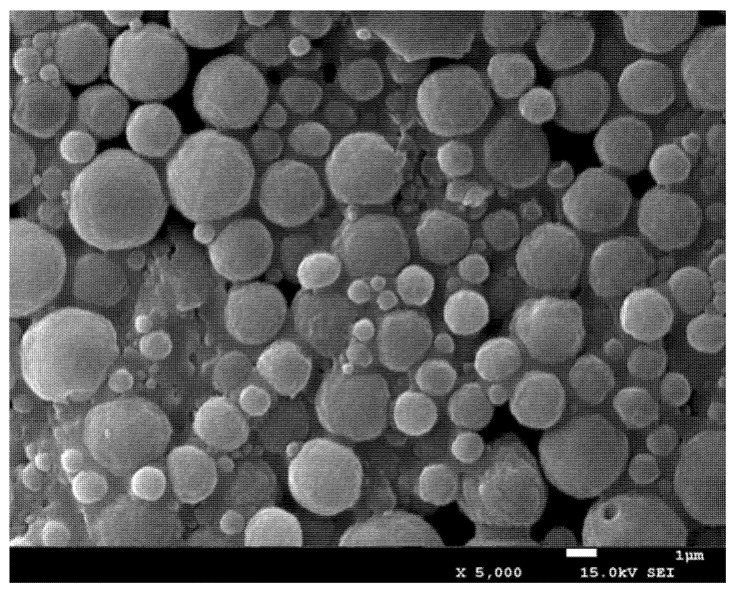
Morphology of MAN-CS-OVA-PLGA-MPs visualized by scanning electron microscopy.

As illustrated in Figure 3, the PLGA-MPs are relatively regular and uniform in size. Compared with PLGA-MPs, mannose-modified CS MPs have partial adhesion, which may be related to the adsorption of CS.

### 3.4. The Particle Size and Electromotive Force of Three Types of PLGA Microspheres

The cell membrane is composed of a phospholipid bilayer structure, with hydrophilic phosphate groups on the outside and hydrophobic fatty acids on the inside. The external phosphate groups and glucuronic acid are negatively charged, and CS is the only natural polysaccharide with a positive charge. As shown in Table 4, the zeta electromotive force (EMF) of OVA-PLGA-MPs was −14.8 MV, while the zeta EMFs of CS-OVA-PLGA-MPs and MAN-CS-OVA-PLGA-MPs increased to 31.6 MV and 30.8 MV, respectively (*p* < 0.05). The particle size of OVA-PLGA-MPs was 6.45 μm, while the CS-OVA-PLGA particle size significantly increased to 9.02 μm (*p* < 0.05). However, the MAN-CS-OVA-PLGA-MPs only increased to 7.9 μm. The drug loading capacities of CS-OVA-PLGA-MPs and MAN-CS-OVA-PLGA-MPs were decreased significantly compared with the OVA-PLGA-MPs. There was no significant difference between the drug loading capacities of CS-OVA-PLGA-MPs and MAN-CS-OVA-PLGA-MPs (*p* > 0.05).

### 3.5. *In Vitro* Experiments

#### 3.5.1. Cytotoxicity of MAN-CS-OVA-PLGA-MPs towards DCs

**Figure 4:**
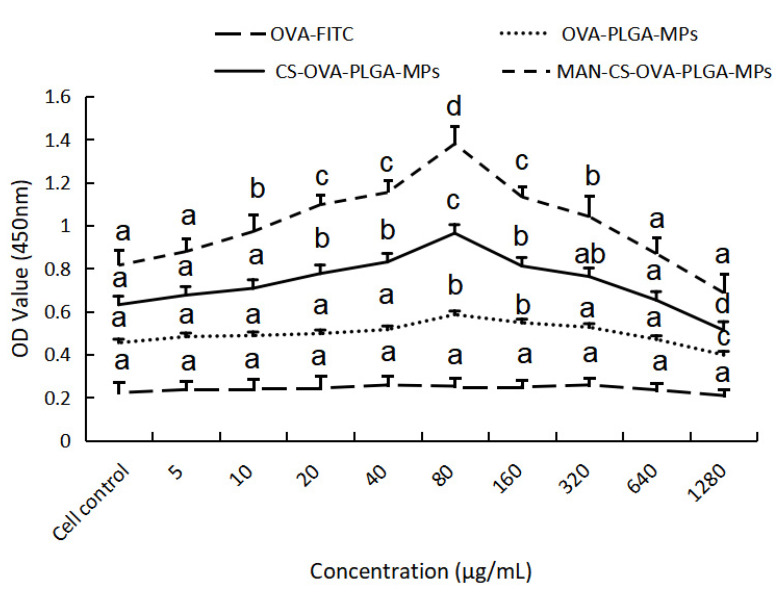
Effect of MAN-CS-OVA-PLGA-MPs on the activity of DCs. Macrophage cell activity was measured using the CCK-8 method. Results are presented as mean ± SD (*n* = 6). Significant differences are indicated with different letters (*p* < 0.05).

As important antigen-presenting cells of the immune system, DCs not only participate in eliciting specific and nonspecific immune responses but also serve as “bridge cells” between the two [34]. Therefore, their normal function will directly or indirectly affect the ability of antigen presentation and clearance during an immune response. The immune-modulation effect of MAN-CS-OVA-PLGA-MPs on DCs is shown in Figure 4. The MAN-CS-OVA-PLGA-MPs were not cytotoxic to DCs in the concentration range of 10–640 μg mL^−1^ and even increased cell proliferation. Maximum cell proliferation activity was observed at a concentration of 80 μg mL^−1^ and was significantly different from the control group (*p* < 0.05). Therefore, a concentration of 80 μg mL^−1^ was selected for subsequent experiments.

#### 3.5.2. Effects of MAN-CS-OVA-PLGA-MPs on the Phagocytic Activity of DCs

**Figure 5:**
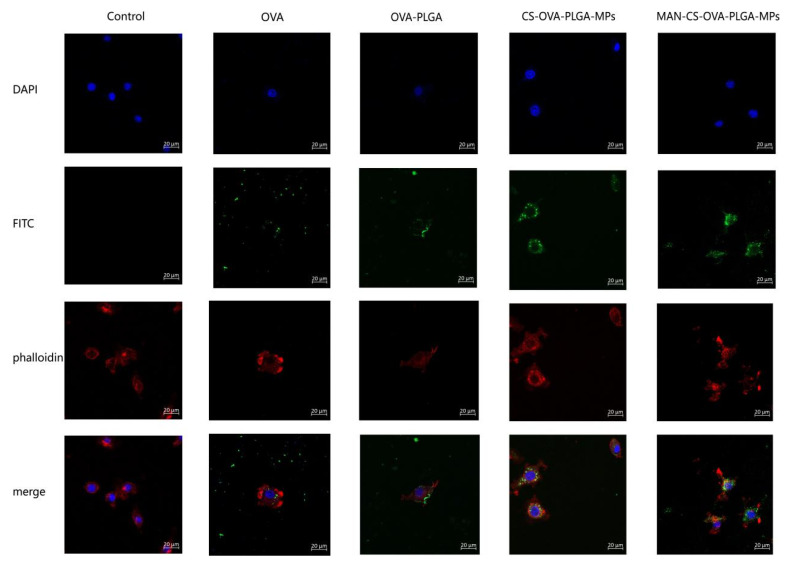
Laser confocal scanning microscopy analysis of MAN-CS-OVA-PLGA-MP uptake by DCs. DCs were inoculated in a 6-well cell-culture plate on a round coverslip. After 24 h of culture, OVA-PLGA, CS-OVA-PLGA-MPs, MAN-CS-OVA-PLGA-MPs, or OVA was added separately. After an additional incubation for 12 h, the cells on the coverslips were fixed and stained using DAPI and Phalloidin-iFluor 555. Blue fluorescence indicates the nucleus, labeled by DAPI, while red fluorescence indicates the actin, stained with Phalloidin-iFluor 555. Cells were mounted with 90% glycerol and visualized using a confocal laser scanning microscope.

In order to explore the ability of DCs to take up MAN-CS-OVA-PLGA-MPs, the phagocytic effect of DCs on antigens was observed using laser confocal scanning microscopy. Different fluorescence intensities indicate the uptake of different antigens by DCs. As shown in Figure 5, the fluorescence intensities in the MAN-CS-OVA-PLGA-MP and CS-OVA-PLGA-MP groups are significantly higher than those in the OVA group. This shows that CS enhanced the ability of the DCs to take up antigens. Compared to the CS-OVA-PLGA-MP group, the fluorescence intensity of the MAN-CS-OVA-PLGA-MP group was significantly increased. We found that MAN-CS-OVA-PLGA-MPs were mainly distributed in the cytoplasm and there was also a large amount of antigen adsorbed on the cell surface to be phagocytosed by DCs. These findings indicate that the MAN-modified CS could enable DCs to take up more OVA.

#### 3.5.3. The Delivery Effect of MAN-CS-OVA-PLGA-MPs on OVA

**Figure 6:**
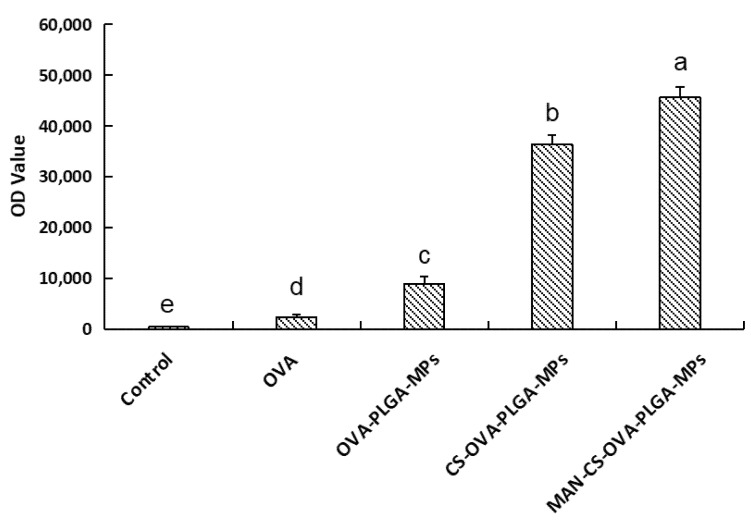
Effect of different microspheres on the phagocytic efficiency of DCs. Results are presented as mean ± SD (*n* = 6). Bars marked with different letters (**a**–**e**) indicate statistically significant differences (*p* < 0.05).

As illustrated in Figure 6, the fluorescence intensity of the MAN-CS-OVA-PLGA-MPs, CS-OVA-PLGA-MPs, and PLGA-MPs was significantly higher than that of the free OVA group (*p* < 0.01). The results show that PLGA displayed passive targeting, which significantly enhanced the efficiency of antigen phagocytosis. In the present study, the fluorescence intensity in the MAN-CS-OVA-PLGA-MP group was significantly higher than that of the CS-OVA-PLGA-MP group (*p* < 0.01). This indicates that the phagocytic effect of the MAN-CS-OVA-PLGA-MPMAN-CS-PLGA MPs group was significantly higher than that of the CS-OVA-PLGA-MP groupCS-PLGA MPs. The MAN-modified CS-conjugated MPs improved the phagocytotic efficiency compared with unmodified CS-conjugated MPs. The glycosylation of MAN promoted active targeting of the microspheres and significantly improved the phagocytosis rate of the MPs.

### 3.6. *In Vivo* Experiments

#### 3.6.1. Effect of MAN-CS-OVA-PLGA-MPs on OVA-Specific IgG and IgG Isotype Antibody Titers of Murine Serum

**Figure 7:**
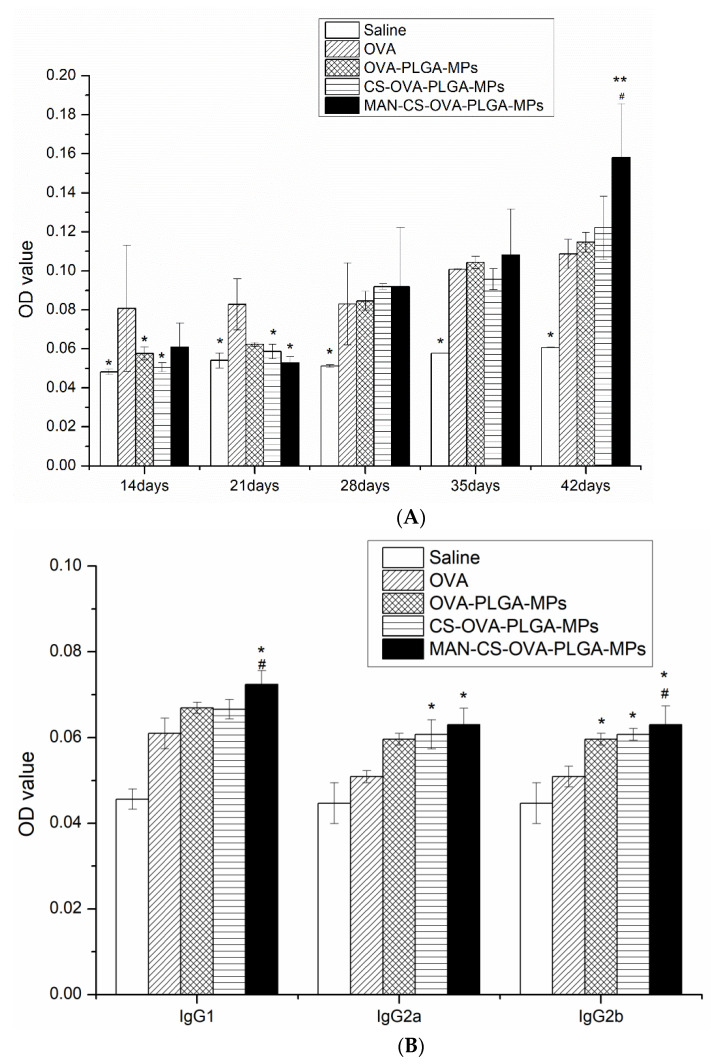
Effect of the MAN-CS-OVA-PLGA-MPs on OVA-specific IgG and IgG subtypes in serum of mice. (**A**) Serum samples were collected for ELISA from immunized ICR mice in all groups between 14–42 days following the first immunization. (**B**) The OVA-specific IgG (A) and IgG isotype (IgG2a, IgG2b, and IgG1) serum levels were determined by ELISA as described in Section 2. The concentrations of IgG and IgG isotopes are presented as mean ± standard deviation. * *p* < 0.05, ** *p* < 0.01 compared with OVA; # *p* < 0.05 compared with CS-OVA-PLGA-MPs. Results are presented as the mean ± SD (*n* = 3).

To evaluate the effect of MAN-CS-PLGA-MPs on serum antibody response in mice, serum was collected, and the OVA-specific IgG antibody was detected by ELISA. As shown in Figure 7A, 14–21 days following the first immunization, the IgG antibody response in the OVA group was higher than that in the other groups, while on day 28, the IgG antibody response induced by injection of MAN-CS-PLGA-MPs began to level off, and on day 42 after the first immunization, the IgG antibody response induced by MAN-CS-PLGA-MPs was significantly higher than that of the OVA group (*p* < 0.01). As illustrated in Figure 7B, the specific IgG1, IgG2a, and IgG2b antibody titers stimulated by MAN-CS-OVA-PLGA-MPs were higher than those in the other groups, and the specific IgG1 and IgG2b produced by mice in the MAN-CS-PLGA-MPs group were significantly higher than those produced by mice in the other groups (*p* < 0.05); compared to OVA-PLGA-MPs alone, the specific IgG2a produced by the mice in MAN-CS-PLGA-MPs group was significantly increased (*p* < 0.05). These data show that MAN-CS-PLGA-MPs could induce a strong, specific humoral immune response.

#### 3.6.2. Effect of MAN-CS-OVA-PLGA-MPs on the Immunophenotype of Spleen Lymphocytes in Mice

**Figure 8:**
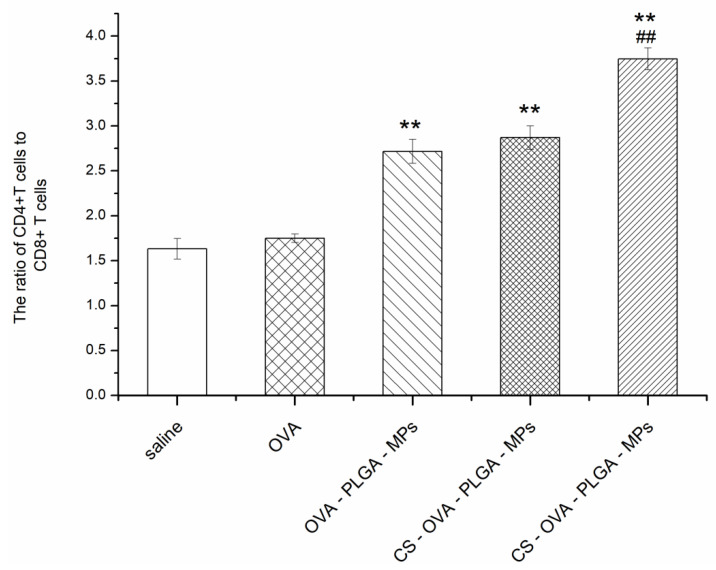
Effects of MAN-CS-OVA-PLGA-MPs on the ratio of CD3^+^ CD4^+^ T cells to CD3^+^ CD8^+^ T cells in spleen cells of mice 28 days following the first immunization. Blood samples were collected and the ratio of CD4^+^/CD8^+^ was determined by FACS. Results are presented as the mean ± SD (*n* = 3). ** *p* < 0.01 compared with OVA group; ## *p* < 0.01 compared with OVA group.

Helper T (Th) cells play a crucial role in the activation of helper B cells and the production of antibodies with high affinity. The CD4 receptor, which is mainly expressed in Th cells, is the common receptor of the T-cell receptor, while the CD8 receptor is expressed in cytotoxic T lymphocytes. The ultimate goal of vaccination is to increase the number of Th cells, improve the immune response, and achieve immune protection and prevention. In this study, the level of Th cells in the spleen lymphocytes of mice immunized with OVA, OVA-PLGA-MPs, CS-OVA-PLGA-MPs, and MAN-CS-OVA-PLGA-MPs was evaluated by flow cytometry. As shown in Figure 8, the proportion of CD4^+^ T cells and CD8^+^ T cells measured in the spleen of mice immunized with PLGA-MPs, CS-OVA-PLGA-MPs, and MAN-CS-OVA-PLGA-MPs was significantly increased compared with the OVA group (*p* < 0.01). Compared with the CS-OVA-PLGA-MPs group, the proportion of CD4^+^ T cells and CD8^+^ T cells in the MAN-CS-OVA-PLGA-MPs group was significantly increased (*p* < 0.01). The present results reveal that both CS-OVA-PLGA-MPs and MAN-CS-OVA-PLGA-MPs promoted an immune response partly by increasing selected T cell subpopulations in immunized mice, with MAN-CS-PLGA-MPs showing a stronger effect than CS-OVA-PLGA-MPs. They also suggest that the MR-targeted nano-MPs can enhance the efficacy of delivery of CS-OVA-PLGA-MPs to T cell subpopulations.

#### 3.6.3. Effect of MAN-CS-OVA-PLGA-MPs on the Expression of DC Surface Molecules in the Spleen

**Figure 9:**
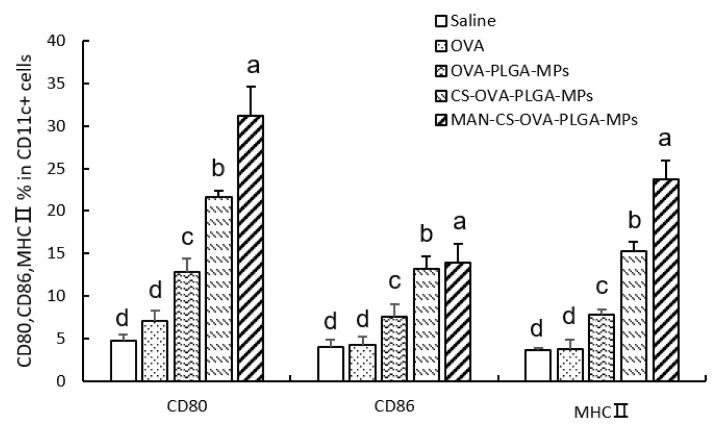
Effect of MAN-CS-OVA-PLGA-MPs on the expression of DC surface molecules in the spleen of mice immunized for 24 h. The spleens were removed from immunized mice on day 3 following the first immunization, and splenocyte suspensions were prepared. The cells were double-stained with anti-CD11c-FITC and anti-MHC II-PE, anti-CD11c-FITC and anti-CD80-PE, or anti-CD11c-FITC and anti-CD86-PE. Then, the expression levels of surface molecules (MHC II, CD80^+^, and CD86^+^) were analyzed using FACS. FITC-stained positive cells represent DC cells. The PE-stained positive cells represent the cells that expressed surface molecules. The PE and FITC double-positive cells identified the DCs that expressed surface molecules. The percentage of PE and FITC double-positive cells were selected and recorded in each sample as well as the percentage of MHC II, CD80^+^, and CD86-positive DCs compared to total DCs. Data are presented as means ± SD (*n* = 3), with different letters (a–d) indicating significant differences (*p* < 0.05).

To evaluate the effect of different MPs on the activation of DCs in mouse spleen, the expression of MHC II, CD80^+^, and CD86^+^ in DCs of the mice was determined by flow cytometry. As shown in Figure 9, CS-OVA-PLGA-MPs and MAN-CS-OVA-PLGA-MPs induced higher CD80^+^, CD86^+^, and MHC II expression 24 h after the first immunization compared with the OVA group. The expression of CD80^+^ and MHC II in the mice of the MAN-CS-OVA-PLGA-MPs group was significantly higher compared with the CS-OVA-PLGA-MPs group (*p* < 0.01). The present data suggest that the use of MAN-CS-OVA-PLGA-MPs significantly improved the maturation of DCs and reveal that the MAN-R-targeted delivery system can efficiently deliver the adjuvant and OVA to DCs, facilitating the maturation of the DCs and enhancing the immune response.

#### 3.6.4. Effect of MAN-CS-OVA-PLGA-MPs on the Concentrations of Cytokines in the Serum of Mice

The concentration of cytokines in the serum of ICR mice was determined by ELISA 42 days following the first immunization. As shown in Table 5, compared with the OVA, CS-OVA-PLGA-MPs, OVA-PLGA-MPs, and saline groups, the concentration of IL-4, IL-6, and IFN-γ in the serum of mice injected subcutaneously with MAN-CS-OVA-PLGA-MPs had increased significantly (*p* < 0.05). MAN-CS-OVA-PLGA-MPs significantly enhanced the production of Th1 and Th2 cytokines in ICR mice, revealing that MAN-CS-OVA-PLGA-MPs can promote both Th1 and Th2 responses simultaneously. The enhancement of cytokine (IL-4, IL-6, and IFN-γ) production with MAN-CS-OVA-PLGA-MPs was stronger than with CS-PLGA-MPs, indicating that the MAN-CS-OVA-PLGA-MPs significantly potentiated the immunological activity of CS-OVA. This also suggests that the MR targeting enhances the delivery efficacy of CS-OVA-PLGA-MPs in mice. The present results demonstrate that MAN-CS-OVA-PLGA-MPs significantly stimulate Th cells to produce Th1- and Th2-type cytokines.

## 4. Discussion

With the research of protein structure and function, many protein drugs have been developed. However, most protein drugs are characterized by a short half-life and poor stability and are readily enzymolyzed. This is a major limitation for their use as therapeutic agents in biomedical research and the pharmaceutical industry. Therefore, the dosage form is single and often requires frequent administration [34]. In recent years, polypeptide and protein drug carriers consisting of biodegradable polymer materials, among which PLGA is the most studied, have attracted the attention of researchers. PLGA has good biocompatibility, its long-term use will not result in accumulation in the body, and it has the characteristics of slow-release drugs, reducing toxicity and side effects [35]. PLGA is a promising base material approved by the FDA and has been used extensively to specifically target drugs to diseases as NPs or as site-specific delivery agents inserted as a film but is limited to loading positively charged molecules [16].

The surface charge of nanoparticles is a crucial biological indicator to influence the interaction between nanoparticles and cells [36]. Although neutral and negatively charged particles may be more advantageous for systemic administration in the blood [36], cationic particles are useful drug carriers, especially for proteins, peptides, and DNA [37,38]. The cell membrane is composed of a phospholipid bilayer structure, with hydrophobic fatty acids on the inside and hydrophilic phosphate groups on the outside. The external phosphate groups and glucuronic acid are negatively charged. Cationic particles can easily interact with the cell membrane and promote subsequent bioactivity. CS is a natural polysaccharide with positively charged cationic polymers [39,40], commonly used as coating materials for biodegradable PLGA or poly (D,L-lactic acid) particles [41] as drug carriers. Cationic surface modification of nanoparticles can be obtained either during the formation of PLGA particles or by incubating polyelectrolytes with preformed PLGA particles [42]. In the present study, we conjugated negatively charged PLGA-MPs to the positively charged MAN-modified CS to form a novel MAN-R-targeted delivery system. Our zeta EMF (mv) results indicate that when OVA-PLGA-MPs were negatively charged, CS-OVA-PLGA-MPs and MAN-CS-OVA-PLGA-MPs became negatively charged as well. This suggested that MAN-modified CS-conjugated antigen loading of PLGA-MPs is an effective strategy to change the surface charge of drug-loading MPs to increase their efficiency of interaction with antigen-presenting cells (APCs).

An ideal vaccine can effectively induce both cellular immunity and humoral immunity, which entails B cell-mediated production of antigen-specific antibodies [43]. In the present study, we demonstrated that MAN-CS-OVA-PLGA-MPs induced mice to produce high levels of IgG, IgG1, IgG2a, and IgG2b. It can be concluded that PLGA-MPs can present antigens more effectively, which can significantly improve the level of specific antibodies and antibody subclasses, and effectively induce a humoral immune response. This finding is consistent with similar previous work, such as that of Li et al. (2015) which studied mannan-decorated thiolated Eudragit MPs (Man-TEM) as a nasal vaccine carrier. The OVA-loaded Man-TEM was used in nasal vaccination in mice, demonstrating that Man-TEM induced higher levels of serum IgG than the soluble OVA group due to the specific recognition of MR of APCs by the mannan part of the Man-TEM, indicating that Man-TEM was sufficient to promote OVA-specific humoral immunity in mice [44].

Cellular immunity is also essential to effective immune-mediated protection against disease, which directs cell-mediated killing and cytokine production enhancing pathogen and tumor cell clearance and regulating system immunity. The spleen is one of the most important immunity organs. Likewise, B cells and T cells are essential immune cells. B cells are mainly involved in humoral immunity and T cells are mainly involved in cellular immunity. Cytokines secreted by Th cells play an important role in regulating the immune response. For example, IFN-γ and IL-2 are mainly secreted and mediated by Th1 cells, while IL-4 and IL-6 are mainly secreted and mediated by Th2 cells. The immune response of cells depends on the activation of antigen-specific CD3^+^CD4^+^ T cells and CD3^+^CD8^+^ T cells [45,46]. CD3 is a surface marker of mature T lymphocytes. Helper T cell 2 (Th2), which has the functions of promoting Th2 cell proliferation, inhibiting Th1 cell proliferation, assisting B cell activation, and enhancing B cell-mediated humoral immunity, is a CD4^+^ T cell. CD8 is mainly expressed on the surface of cytotoxic T cells (CTLs) and can kill target cells specifically [47]. The mutual regulation of immune responses depends on the exchange of cytokines secreted by various types of Th cells to ensure the normal signal transmission and maintain the stability of the immune inner ring [48]. The ultimate goal of vaccination is to improve the immune enhancement effect of Th cells, promote the activation of B cells to produce antibodies, and achieve the purpose of immune protection and disease prevention [48]. In the present study, the ratio of CD3^+^CD4^+^/CD3^+^CD8^+^ T cells in spleen lymphocytes of immunized mice was detected by flow cytometry. The results show that the ratio of CD3^+^CD4^+^/CD3^+^CD8^+^ T cells was highest in mice immunized using MAN-CS-OVA-PLGA-MPs. In this study, we also determined the concentration of Th1 and Th2 cytokines in the serum of immunized mice and found that MAN-CS-OVA-PLGA-MPs can promote the secretion of cytokines by two types of cells, which shows that the application of MAN-CS-OVA-PLGA-MPs as an antigen delivery system can effectively stimulate the immune activity of Th cells.

Immunotherapy refers to the treatment of a low or high immune state of the body, artificially enhancing or inhibiting the immune function of the body in order to achieve the purpose of disease treatment [49]. Immunotherapy requiring an efficient T lymphocyte response is initiated by antigen delivery to APCs. DCs are the most effective “professional” APCs. Several key steps are involved in the induction of the protective immune response, including the uptake of APCs, the treatment with antigens, and the activation of APCs to effectively activate T cells and B cells [49,50]. In addition, DCs are the main regulator of the adaptive immune response and the only cell type that can promote the proliferation of T cells [51]. Immature DCs have a strong endocytic ability and express various pathogen-recognition receptors, such as the toll-like receptor (TLR), and continuously collect danger signals from their surroundings. TLR triggers phenotypic changes, promotes Ag processing and MHC presentation, and increases cytokine production, a process known as DC maturation [52,53]. Immature DCs are transformed into mature DCs that express relatively high levels of surface MHC (class I and class II) and costimulatory molecules, including CD80^+^ and CD86^+^. Upon maturation, the antigen uptake ability of DC cells decreases, but their T cell stimulation ability increases, which increases the chance of T cell capture and interaction [54]. Therefore, the expression of surface molecules indicates the maturation and activation of DCs, which is a prerequisite for effective antigen presentation. Three kinds of MPs were prepared in this work and used to immunize mice by injection. The expression level of DC surface molecules in spleen cells of the immunized mice in each group was measured 24 h following injection. The present results indicate that MAN-CS-PLGA-MPs significantly promoted the expression of CD80^+^, CD86^+^, and MHC II in mice spleen DCs cells, which confirmed that MAN-CS-PLGA-MPs more strongly activated the maturation of DCs compared with the other MPs. The maturation level of DCs in the spleen provides excellent conditions for the activation of effector T cells and for the stimulation of the cellular immune response [55,56].

## 5. Conclusions

In this work, we showed that MAN-CS-OVA-PLGA-MPs acted as an MR-targeting antigen delivery system that significantly enhanced the cellular and humoral immune response via facilitating the maturation of DCsRAW264.7. Our results reveal that MAN-CS-OVA-PLGA-MPs efficiently delivered the CS and OVA to DCs and significantly promoted the antigen-presenting efficiency. An immunization strategy using MAN-CS-OVA-PLGA-MPs encapsulating antigen or adjuvant is an efficient approach for antigen presentation that induces stronger antigen-specific immune responses than free antigen. These findings pave the way to using MAN-CS-OVA-PLGA-MPs as an antigen and adjuvant delivery system for future vaccine formulations and to delivering nanotherapeutics to diseased cells, improving their potency and efficacy.

## Figures and Tables

**Table 1:** Factors and corresponding levels for experimental design and optimization.

	PLGA	PVA	OVA
1	10	1	50
2	20	2	100
3	40	3	150

**Table 2:** The results of elemental analysis.

Chitosan	Mannose-Modified Chitosan
C:39.99	C:39.26
H:6.42	H:6.87
O:45.37	O:45.52
N:7.63	N:7.24

**Table 3:** L9 (3^3^) orthogonal table.

	PLGA	PVA	OVA	LE(%)
1	10	1	50	3.25
2	10	2	100	3.15
3	10	3	150	1.27
4	20	1	100	1.69
5	20	2	150	6.48
6	20	3	50	3.22
7	40	1	150	5.83
8	40	2	50	8.4
9	40	3	100	8.1
K1	2.55	3.59	4.95	
K2	3.79	6.01	4.31	
K3	7.44	4.19	4.52	
R	4.88	2.42	0.43	
	A3B2C1			

**Table 4:** Physical properties of three different types of PLGA microspheres.

Group	Zeta EMF (mv)	Particle Size (μm)	Drug Loading (%)
OVA-PLGA-MPs	−14.8 ± 1.9 ^a^	6.5 ± 0.4 ^a^	7.9 ± 0.2 ^a^
CS-OVA-PLGA-MPs	31.6 ± 0.4 ^b^	9.0 ± 0.5 ^b^	6.5 ± 0.2 ^b^
MAN-CS-OVA-PLGA-MPs	30.8 ± 1.1 ^b^	7.9 ± 0.5 ^c^	6.2 ± 1.2 ^b^

Results are presented as the mean ± SD. Data within a column without the same superscripts ^(a–^^c)^ differ significantly (*n* = 3, *p* < 0.05).

**Table 5:** Effect of MAN-CS-OVA--PLGA-MPs on the level of several cytokines in the serum of mice.

Group	IL-2 (pg/mL)	IL-4 (pg/mL)	IL-6 (pg/mL)	IFN-γ (pg/mL)
Saline	4.295 ± 0.045 ^a^	11.540 ± 2.820 ^a^	36.835 ± 0.485 ^a^	27.200 ± 0.630 ^a^
OVA	4.210 ± 0.580 ^a^	12.735 ± 0.835 ^a,b^	37.320 ± 1.230 ^a^	28.665 ± 0.845 ^a,b^
OVA-PLGA-MPs	4.245 ± 0.315 ^a^	16.015 ± 2.845 ^a,b^	37.805 ± 1.275 ^a,b^	28.900 ± 0.430 ^a,b^
CS-OVA-PLGA-MPs	4.525 ± 0.165 ^a^	16.255 ± 1.185 ^b^	39.930 ± 0.750 ^b^	29.565 ± 0.075 ^a,b^
MAN-CS-OVA-PLGA-MPs	4.635 ± 0.045 ^a^	21.860 ± 3.720 ^c^	40.175 ± 1.975 ^c^	30.525 ± 1.065 ^b^

Data are means ± S.D. (*n* = 3). Data within a column without the same superscripts (a–c) differ significantly (*p* < 0.05).

## References

[1] Hannah B.M., Joanna L.T., Mats A., Ed C.L. (2018). Immunomodulatory properties of *chitosan* polymers. Biomaterials.

[2] Muxika A., Etxabide A., Uranga J., Guerrero P., de la Caba K. (2017). *Chitosan* as a bioactive polymer: Processing, *properties* and applications. Int. J. Biol. Macromol..

[3] Wang Q., Zhao Y., Guan L., Zhang Y., Dang Q., Dong P., Li J., Liang X.G. (2017). Preparation of astaxanthin-loaded DNA/chitosan nanoparticles for improved cellular uptake and antioxidation capability. Food Chem..

[4] Gabriel P.M., Ignacimuthu S., RajivGandhi M., Shajahan A., Ganesan P. (2017). Comparative studies of tripolyphosphate and glutaraldehyde cross-linked chitosan-botanical pesticide nanoparticles and their agricultural applications. Int. J. Biol. Macromol..

[5] Saad Q., Muhammad Z., Shariq N., Khurshid Z., Shah A., Husain S., Shehriar H., Ihtesham R. (2018). Electrospinning of Chitosan-Based Solutions for Tissue Engineering and Regenerative Medicine. Int. J. Mol. Sci..

[6] Zheng Y., Yan S., Shi B. (2018). Mechanism of antioxidative stress and anti-inflammatory action of chitosan and its derivatives. J. Anim. Nutr..

[7] Shi G.N., Zhang C.N., Xu R., Niu J.F., Song H.J., Zhang X.Y., Wang W.W., Wang Y.M., Li C., Wei X.Q. (2016). Enhanced antitumor immunity by targeting dendritic cells with tumor cell lysate-loaded chitosan nanoparticles vaccine. Biomaterials.

[8] Wang X., Zhang W., Liu F., Zheng M., Zheng D., Zhang T. (2012). Intranasal immunization with live attenuated influenza vaccine plus chitosan as an adjuvant protects mice against homologous and heterologous virus challenge. Arch. Virol..

[9] Tao W., Zheng H.Q., Fu T. (2017). N-(2-hydroxy) propyl-3-trimethylammonium chitosan chloride: An immune-enhancing adjuvant for hepatitis E virus recombinant polypeptide vaccine in mice. Hum. Vaccine Immunother..

[10] Farris E., Brown D., Ramer-Tait A., Pannier A. (2017). Chitosan-zein nano-in-microparticles capable of mediating in vivo transgene expression following oral delivery. J. Control. Release.

[11] Wu J., Wu N., Yue H. (2016). Safety evaluation of chitosan quaternary ammonium salt microspheres as adjuvant for injection vaccine. J. Process Eng..

[12] Prajapati S., Jain A., Jain A., Jain S. (2019). Biodegradable polymers and constructs: A novel approach in drug delivery. Eur. Polym. J..

[13] Ghitman J., Biru E., Stan R., Iov H. (2020). Review of hybrid PLGA nanoparticles: Future of smart drug delivery and theranostics medicine. Mater. Des..

[14] Wu X.Y., Li Y.C., Raza F., Wang X.R., Zhang S.L., Rong R.N., Qiu M.F., Su J. (2021). Red Blood Cell Membrane-Camouflaged Tedizolid Phosphate-Loaded PLGA Nanoparticles for Bacterial-Infection Therapy. Pharmaceutics.

[15] Cheng S., Nethi S.K., Al-Kofahi M., Prabha S. (2021). Pharmacokinetic—Pharmacodynamic Modeling of Tumor Targeted Drug Delivery Using Nano-Engineered Mesenchymal Stem Cells. Pharmaceutics.

[16] Duskey J.T., Baraldi C., Gamberini M.C., Ottonelli I., Ros F.D., Tosi G., Forni F., Vandelli M.A., Ruozi B. (2020). Investigating Novel Syntheses of a Series of Unique Hybrid PLGA-Chitosan Polymers for Potential Therapeutic Delivery Applications. Polymers.

[17] George A., Shah P., Shrivastav P. (2019). Natural biodegradable polymers-based nano-formulations for drug delivery: A review. Int. J. Pharm..

[18] Lima A.F., Amado I.R., Pires L.R. (2020). Poly(d, l-lactide-co-glycolide) (PLGA) Nanoparticles Loaded with Proteolipid Protein (PLP)—Exploring a New Administration Route. Polymers.

[19] Khan A., Zhou Z.W., He W.M., Gao K., Khan M.W., Faisal R., Muhammad H., Sun M. (2019). CXCR4-Receptor-Targeted Liposomes for the Treatment of Peritoneal Fibrosis. Mol. Pharm..

[20] Luo L., Zheng S., Huang Y., Qin T., Xing J., Niu Y., Bo R., Liu Z., Huang Y., Hu Y. (2016). Preparation and characterization of Chinese yam polysaccharide PLGA nanoparticles and their immunological activity. Int. J. Pharm..

[21] Raza F., Zafar H., Zhang S.L., Kamal Z., Su J., Yuan W.E., Qiu M.M. (2021). Recent Advances in Cell Membrane-Derived Biomimetic Nanotechnology for Cancer Immunotherapy. Adv. Healthc. Mater..

[22] Phanthipha R., Peerapong P., Prapaporn U. (2017). A mannose-specific C-type lectin from Fenneropenaeus merguiensis exhibited antimicrobial activity to mediate shrimp innate immunity. Mol. Immunol..

[23] Raza F., Zafar H., Zhu Y., Ren Y., Ullah A., Khan A.U., He X.Y., Han H., Aquib M., Boakye-Yiadom K.O. (2018). Review on Recent Advances in Stabilizing Peptides/Proteins upon Fabrication in Hydrogels from Biodegradable Polymers. Pharmaceutics.

[24] Chen L. (2009). Construction and Evaluation of Dendritic Cell Targeting Vector Mediated by Mannose Receptor.

[25] Feng J., Zhao L., Yu Q. (2004). Receptor-mediated stimulatory effect of oligochitosan in macrophages. Biochem. Biophys. Res. Commun..

[26] Yamashita M., Otsuka F., Mukai T., Otani H., Inagaki K., Miyoshi T. (2008). TNF-α inhibition of BMP-2-induced osteoblast differentiation by regulating Smad signaling and Ras/Rho-MAPK pathway. Clin. Chim. Acta..

[27] Wi T.I., Byeon Y., Won J.E., Lee J.M., Kang T.H., Lee J.-W., Lee Y.J., Sood A.K., Han H.D., Park Y.M. (2020). Selective Tumor-Specific Antigen Delivery to Dendritic Cells Using Mannose-Labeled Poly(d, l-lactide-co-glycolide) Nanoparticles for Cancer Immunotherapy. J. Biomed. Nanotechnol..

[28] Zhu J.H., Qin F.H., Ji Z.H., Fei W., Tan Z., Hu Y., Zheng C.H. (2019). Mannose-Modified PLGA Nanoparticles for Sustained and Targeted Delivery in Hepatitis B Virus Immunoprophylaxis. AAPS Pharm. Sci. Tech..

[29] Feng H.B., Fan J., Song Z.H., Du X.G., Chen Y., Wang J.S., Song G.D. (2016). Characterization and immunoenhancement activities of Eucommia ulmoides polysaccharides. Carbohydr. Polym..

[30] Feng H.B., Fan J., Lin L., Liu Y.J., Chai D.K., Yang J. (2019). Immunomodulatory Effects of Phosphorylated Radix Cyathulae officinalis Polysaccharides in Immunosuppressed Mice. Molecules.

[31] Feng H.B., Fan J., He B., Xi T., He B., Wang X. (2016). Selenylation modification can enhance immune-enhancing activity of Chuanminshen violaceum polysaccharide. Carbohydr. Polym..

[32] Feng H.B., Du X.G., Liu J., Han X.F., Cao X.H., Zeng X.Y. (2014). Novel polysaccharide from Radix Cyathulae officinalis Kuan can improve immune response to ovalbumin in mice. Int. J. Biol. Macromol..

[33] Zhou X., Liu B., Yu X., Zha X., Zhang X., Chen Y. (2007). Controlled release of PEI/DNA complexes from mannose bearing chitosan microspheres as a potent delivery system to enhance immune response to HBV DNA vaccine. J. Control. Release.

[34] Zhang C.Q., Yang L., Wan F., Bera H., Cun D.M., Rantanen J., Yang M. (2020). Quality by design thinking in the development of long-acting injectable *PLGA*/PLA-based microspheres for peptide and protein drug delivery. Int. J. Pharm..

[35] Sun M., Ban J., Huang S. (2011). Influencing factors and control of drug loading and entrapment rate of PLGA microspheres. J. Guangdong Inst. Pharm..

[36] Ulusoy A., Onur M.A. (2003). Measurement of in vitro phagocytic activity using functional groups carrying monodisperse poly(glycidyl methacrylate) microspheres in rat blood. J. Biomater. Sci. Polym. Ed..

[37] Singh M., Kazzaz J., Ugozzoli M., Chesko J., O’Hagan D.T. (2004). Charged polylactide co-glycolide microparticles as antigen delivery systems. Expert Opin. Biol..

[38] Mandal B., Kempf M., Merkle H.P., Walter E. (2004). Immobilisation of gm-csf onto particulate vaccine carrier systems. Int. J. Pharm..

[39] Kumar M., Bakowsky U., Lehr C.M. (2004). Preparation and characterization of cationic plga nanospheres as DNA carriers. Biomaterials.

[40] Kumar M.N., Mohapatra S.S., Kong X., Jena P.K., Bakowsky U., Lehr C.M. (2004). Cationic poly(lactide-coglycolide) nanoparticles as efficient in vivo gene transfection agents. J. Nanosci. Nanotechnol..

[41] Munier S., Messai I., Delair T., Verrier B., Ataman-Onal Y. (2005). Cationic pla nanoparticles for DNA delivery:Comparison of three surface polycations for DNA binding, protection and transfection properties. Colloids Surf. B Biointerfaces.

[42] Tsung M.J., Burgess D.J. (2001). Preparation and characterization of gelatin surface modified plga microspheres. AAPS PharmSci.

[43] Darrell J.I., Benjamin J.R. (2020). Shaping *humoral immunity* to *vaccines* through antigen-displaying nanoparticles. Curr. Opin. Immunol..

[44] Li H., Tae-EunPark B., Hong Z., Kang S., Cho C., Choi Y. (2015). Mannan-decorated thiolated Eudragit microspheres for targeting antigen presenting cells via nasal vaccination. Eur. J. Pharm. Sci..

[45] Benjamin G.K., Abhinav A., Jamal S.L. (2020). *Innate* and *Adaptive Immunity*: The *Immune* Response to Foreign Materials. Biomater. Sci..

[46] Singh R., Alape D., de Lima A., Ascanio J., Majid A., Gangadharan S.P. (2019). Regulatory T Cells in Respiratory Health and Diseases. BMC Pulm. Med..

[47] Saigusa R., Winkels H., Ley K. (2020). T cell subsets and functions in atherosclerosis. Nat. Rev. Cardiol..

[48] Reddy S., Vlies A., Simeoni E., Angeli V., Randolph G., Neil C. (2007). Exploiting lymphatic transport and complement activation in nanoparticle vaccines. Nat. Biotechnol..

[49] Constantino J., Gomes C., Falcão A., Neves B.M., Cruz M.T. (2017). Dendritic cell-based immunotherapy: A basic review and recent advances. Immunol. Res..

[50] Christian E.B., Sarah S., Benjamin K., Michael S.P., Phillip D.F., Derek N.J.H. (2019). *Dendritic* cells as *cancer* therapeutics. Semin. Cell Dev. Biol..

[51] Hartgers F., Figdor C., Adema G. (2000). Towards a molecular understanding of dendritic cell immunobilogy. Immunol. Today.

[52] Wculek S.K., Cueto F.J., Mujal A.M., Melero I., Krummel M.F., Sancho D. (2020). Dendritic cells in cancer immunology and immunotherapy. Nat. Rev. Immunol..

[53] Boraschi D., Italiani P., Palomba R., Decuzzi P., Duschl A., Fadeel B., Moghimi S.M. (2017). Nanoparticles and innate immunity: New perspectives on host defence. Semin Immunol..

[54] Dowling J.K., Mansell A. (2016). Toll-like receptors: The swiss army knife of immunity and vaccine development. Clin. Transl. Immunol..

[55] Gardner A., de Mingo Pulido Á., Ruffell B. (2020). Dendritic Cells and Their Role in Immunotherapy. Front. Immunol..

[56] Keselowsky B.G., Lewis J.S. (2017). Dendritic cells in the host response to implanted materials. Semin Immunol..

